# Effect of Fasting Blood Glucose Level on Heart Rate Variability of Healthy Young Adults

**DOI:** 10.1371/journal.pone.0159820

**Published:** 2016-07-21

**Authors:** Mohamed Faisal Lutfi, Ramaze Farouke Elhakeem

**Affiliations:** Department of Physiology, Faculty of Medicine and Health Sciences, Al-Neelain University, Khartoum, Sudan; Weill Cornell Medical College Qatar, QATAR

## Abstract

**Background:**

Previous studies reported increased risk of cardiac events in subjects with fasting blood glucose (FBG) levels lower than the diagnostic threshold of diabetes mellitus. However, whether increased cardiac events in those with upper normal FBG is secondary to the shift of their cardiac sympathovagal balance towards sympathetic predominance is unknown.

**Aims:**

To assess the association between FBG levels and cardiac autonomic modulation (CAM) in euglycaemic healthy subjects based on heart rate variability (HRV) derived indices.

**Subjects and Methods:**

The study enrolled 42 healthy young adults. Following sociodemographic and clinical assessment, blood samples were collected to measure FBG levels. Five minutes ECG recordings were performed to all participants to obtain frequency domain HRV measurements, namely the natural logarithm (Ln) of total power (LnTP), very low frequency (LnVLF), low frequency (LnLF) and high frequency (LnHF), low frequency/ high frequency ratio (LnLF/HF), normalized low frequency (LF Norm) and high frequency (HF Norm).

**Results:**

FBG levels correlated positively with LnHF (*r* = 0.33, P = 0.031) and HF Norm (*r* = 0.35, P = 0.025) and negatively with LF Norm (*r* = -0.35, P = 0.025) and LnLF/HF (*r* = -0.33, P = 0.035). LnHF and HF Norm were significantly decreased in subjects with the lower (4.00 (1.34) ms^2^/Hz and 33.12 (11.94) n.u) compared to those with the upper FBG quartile (5.64 (1.63) ms^2^/Hz and 49.43 (17.73) n.u, P = 0.013 and 0.032 respectively). LF Norm and LnLF/HF were significantly increased in subjects with the lower (66.88 (11.94) n.u and 0.73 (0.53)) compared to those with the higher FBG quartile (50.58 (17.83) n.u and 0.03 (0.79), P = 0.032 and 0.038 respectively).

**Conclusion:**

The present study is the first to demonstrate that rise of blood glucose concentration, within physiological range, is associated with higher parasympathetic, but lower sympathetic CAM. Further researches are needed to set out the glycemic threshold beyond which further increase in glucose level readjusts sympathovagal balance towards sympathetic predominance again.

## Introduction

Currently, heart rate variability (HRV) is among the useful tools used to assess modulatory effects of the autonomic nervous system on the heart [1]. From physiological standpoint, the interaction between the sympathetic and parasympathetic discharge to the heart is a major determinant of HRV and consequently cardiac autonomic modulations (CAM) [1–3]. Time and frequency domains analyses are commonly used to evaluate HRV and CAM; however, frequency domain is preferred if HRV measurements are derived from short-term (5-minute) ECG recording [4]. Natural logarithm (Ln) of total power (LnTP), very low frequency (LnVLF), low frequency (LnLF) and high frequency (LnHF), low frequency/ high frequency ratio (LnLF/HF), normalized low frequency (LF Norm) and high frequency (HF Norm) are the main measurements used to express results of frequency domain HRV analysis [4–6]. LnTP truly reflect the overall HRV [4]. LnVLF and LnHF are currently believed to be influenced by parasympathetic regulation; however, there are many concerns about the physiological basis of LnVLF [4, 5]. Although LnLF was previously used as an indicator of sympathetic modulations, recent studies demonstrate it as a parasympathetic index [7, 8]. LF Norm and HF Norm are commonly used to assess modulatory effects of sympathetic and parasympathetic discharge on the heart respectively [6, 9]. LnLF/HF is used as a measure of sympathovagal balance [4]. Noteworthy, mean heart rate (MHR) during the period of ECG recording is inversely related HRV [4, 5] and should be adjusted for if CAM is compared between two groups with different MHR [6, 9].

Most previous studies utilized HRV in risk stratification of cardiac events especially following myocardial infarction [10, 11]. In clinical practice, augmented sympathetic CAM is associated with low HRV and increased the risk of cardiac events among susceptible patients [4, 5]. Recent researches used HRV to estimate the impact of autonomic dysfunction on certain cardiovascular risk factors like hypertension [12], diabetes mellitus [13] and others [1, 4, 5]. Poor HRV and higher sympathetic activity were demonstrated in hypertensive patients [12] and subjects with high normal readings of blood pressures [14]. Similar trends of HRV and CAM were demonstrated in cases with hyperglycemia [13, 15] as well as hypoglycemia [16, 17]. However, whether these trends extend to the normal physiological levels of blood glucose are not clear. There are accumulating evidences that cardiovascular events are higher at fasting [18] or 2-hours [19] glucose levels lower than the diagnostic threshold for diabetes mellitus. A prospective study confirmed that subjects with fasting blood glucose [FBG] levels > 85 mg/dl had significantly increased cardiovascular mortality compared with those with lower levels [18]. This finding raises a question whether increased cardiovascular mortality at the upper limits of normal FBG range is associated with shift of cardiac sympathovagal balance towards sympathetic predominance. For further exploration of this opinion, we presently designed this study to assess the association between FBG levels and CAM in euglycaemic healthy subjects based on HRV-derived indices.

## Materials and Methods

The present study received ethical approval from ethics review committee (ERC), Faculty of Medicine, University of Khartoum, Sudan. All subjects who were willing to participate in the study signed a written informed consent before being examined.

The study enrolled 42 young adults (23 males and 19 females). All volunteers were medical students or staff members at Alneelain University, Khartoum, Sudan. The selection criteria were: interest to participate in the study, age of 20–40 years, absence of chronic diseases including diabetes mellitus, hypertension, thyroid dysfunction, heart diseases or any illness known alter heart rhythm. None of the studied subjects were on regular medication. Sociodemographic characteristics, past medical history and clinical examination were collected guided by a questionnaire. The body mass index (BMI) was estimated by subdividing the weight in kilograms by the height in squared meters. The mean arterial blood pressure (MABP) was calculated by adding the diastolic blood pressure to one third of the difference between the systolic and the diastolic blood pressures. FBG levels were measured in all volunteers, after ensuring an overnight fasting of at least 8 hours, using the calorimetric method (JENWAY 6051 Colorimeter, Bibby Scientific Limited, UK). FBG levels of all studied subjects were below diabetic threshold (< 110 mg/dl).

Using Heart Rhythm Scanner (Version 2.0, Biocom Technologies, Poulsbo, WA, U.S.A), frequency domain HRV indices were measured based on supine, 5-min ECG recording. ECG recordings were conducted in all participants during the period from 09.00 AM–12.00 AM to guard against possible circadian effects on HRV parameters. Clear ECG signals, lack of ECG movement artifacts and easy regular breathing were ensured during ECG recording. Frequency domain HRV measurements, namely LnTP, LnVLF, LnLF, LnHF, LF Norm, HF Norm and LnLF/HF, were used to interpret HRV and CAM among the studied subjects as previously described [1, 4–6, 9]. The Heart Rhythm Scanner also calculated MHR during the period of ECG recoding.

Data were entered into the computer via SPSS for Windows (Version 16; Chicago, IL, USA). Studied variables were described with means (M) and standard deviations (SD). Quintiles were used to create cut-off points for FBG levels among the studied subjects. To guard against possible confounding effects of heart rate on indicators of cardiac autonomic modulations [20, 21], comparable distributions MHR were ensured among different quintiles using one way ANOVA and LSD Post Hoc analysis. Associations between FBG levels and frequency domain HRV indices were assessed with bivariate correlations. Student T-test was used to assess the differences in the means of HRV parameters between the upper and lower FBG quintiles as well as between the upper quintile and the mean of the remaining four FBG quintiles. Linear regression analysis was used to assess possible influences of FBG level, gender, age, BMI, MHR and MABP on LnHF, HF Norm, LF Norm and LnLF/HF. P < 0.05 was considered statistically significant.

## Results

The M (SD) of age, BMI, FBG level, MABP, MHR and frequency domain HRV parameters are given in Table 1.

**Table 1:** Characteristics of studied individuals.

	M (SD)
	N = 42
Age (Years)	25.83 (4.83)
BMI (Kg/m2)	24.34 (5.63)
FBG level (mg/dl)	87.21 (9.16)
MABP (mmHg)	90.40 (14.64)
MHR (Beat/Minute)	81.89 (10.60)
LnTP (ms^2^/Hz)	6.67 (1.31)
LnVLF (ms^2^/Hz)	5.51 (1.22)
LnLF (ms^2^/Hz)	5.47 (1.36)
LnHF (ms^2^/Hz)	5.29 (1.70)
LF Norm (n.u)	54.07 (17.93)
HF Norm (n.u)	45.94 (17.94)
LnLF/HF	0.18 (0.79)

FBG levels correlated positively with LnHF (*r* = 0.33, P = 0.031) and HF Norm (*r* = 0.35, P = 0.025) and negatively with LF Norm (*r* = -0.35, P = 0.025) and LnLF/HF (*r* = -0.33, P = 0.035), Table 2.

**Table 2:** Correlations between FBG levels and frequency domain HRV indices.

	*r*	P
LnTP (ms^2^/Hz)	0.26	0.098
LnVLF (ms^2^/Hz)	0.18	0.265
LnLF (ms^2^/Hz)	0.22	0.164
LnHF (ms^2^/Hz)	0.33	0.031
LF Norm (n.u)	-0.35	0.025
HF Norm (n.u)	0.35	0.025
LnLF/HF	-0.33	0.035

**Fig 1:**
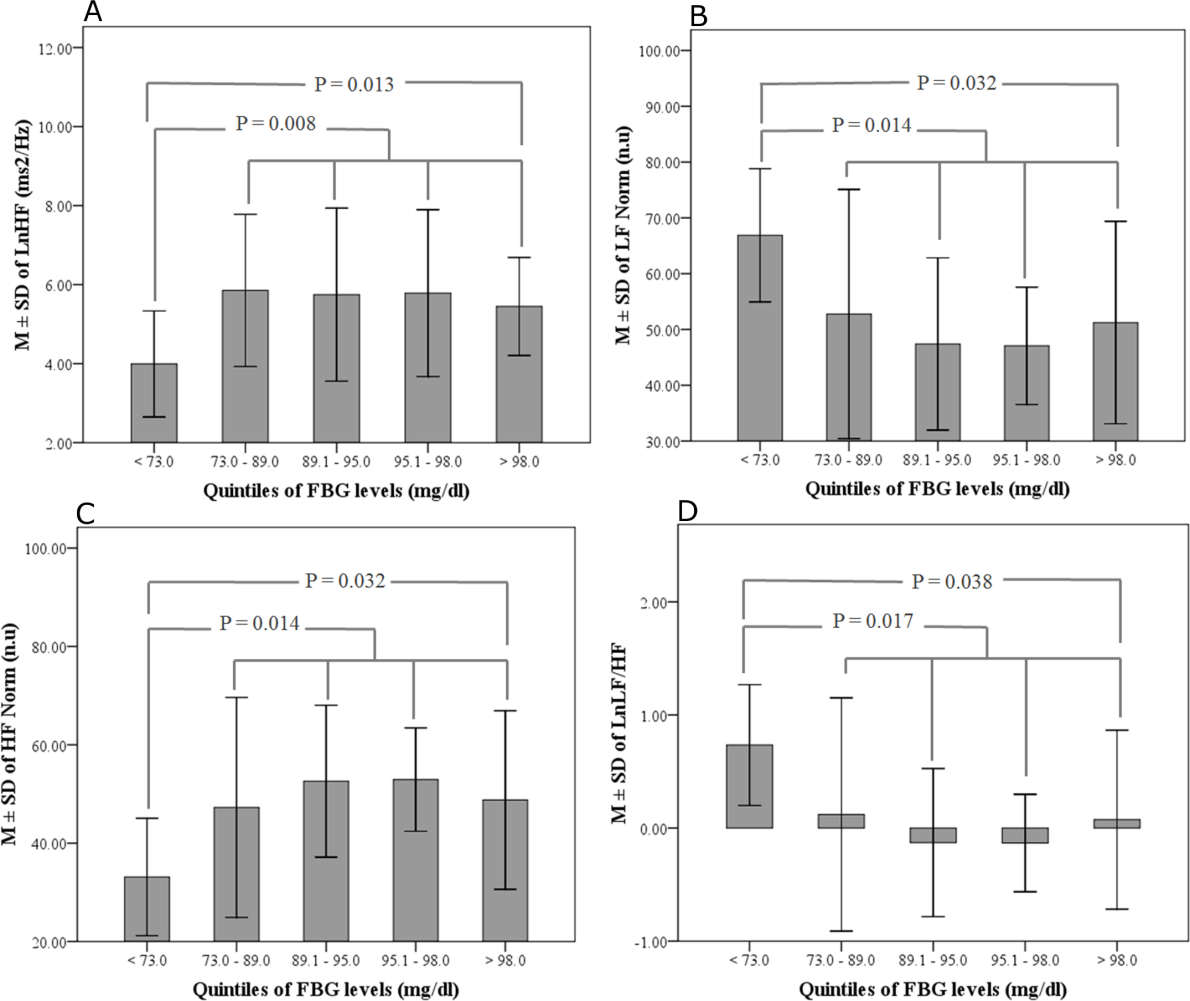
M±SD of (A) LnHF, (B) LF Norm, (C) HF Norm and (D) LnLF/HF Norm in subjects grouped according to FBG quintiles. The P values refer to the significant difference of the respective HRV indices when subjects with lower and upper FBG quartiles, or lower and the mean of all other FBG quintiles, were compared using Student T-test.

Quintiles cut-off points for FBG levels were < 73.0 mg/dl, 73.0–89.0 mg/dl, 89.1–95.0 mg/dl, 95.1–98.0 mg/dl and > 98.0 mg/dl. There were no significant differences in the MHR between the studied subjects when grouped according to FBG quintiles (F = 1.91, P = 0.331). LnHF and HF Norm were significantly decreased in subjects with the lower (4.00 (1.34) ms^2^/Hz and 33.12 (11.94) n.u) compared to those with the upper FBG quartile (5.64 (1.63) ms^2^/Hz and 49.43 (17.73) n.u, P = 0.013 and 0.032 respectively). In contrast, LF Norm and LnLF/HF were significantly increased in subjects with the lower (66.88 (11.94) n.u and 0.73 (0.53)) compared to those with the higher FBG quartile (50.58 (17.83) n.u and 0.03 (0.79), P = 0.032 and 0.038 respectively). Similar pattern of changes were noted for LnHF, HF Norm, LF Norm and LnLF/HF when those with the lower FBG quintile were compared with all other studied subjects, Fig 1.

Gradual decrease in LnHF and HF Norm and simultaneous increase in LF Norm and LnLF/HF were noted in the upper two FBG quintiles (Fig 1); however, Post Hoc analysis failed to document statistical significant for this rise.

Linear regression analysis confirmed that influence of FBG level on LnHF, HF Norm, LF Norm and LnLF/HF is independent of gender, age, BMI, MHR or MABP of the studied subjects, Table 3.

**Table 3:** Linear regression analysis of factors associated with LnHF, LF Norm, HF Norm and LnLF/HF.

	LnHF			LF Norm			HF Norm			LnLF/HF		
	CE	SE	P	CE	SE	P	CE	SE	P	CE	SE	P
Gender	0.05	0.50	0.927	10.1	5.39	0.069	-10.1	5.39	0.069	0.44	0.24	0.076
Age (Years)	-0.02	0.05	0.647	0.02	0.57	0.971	-0.02	0.57	0.969	0.00	0.03	0.954
BMI (Kg/m2)	-0.06	0.05	0.209	0.36	0.52	0.497	-0.36	0.52	0.499	0.01	0.02	0.601
MHR (Beat/Minute)	-0.02	0.02	0.507	-0.11	0.26	0.675	0.11	0.26	0.675	0.00	0.01	0.706
MABP (mmHg)	-0.05	0.03	0.116	0.48	0.31	0.134	-0.48	0.31	0.133	0.02	0.01	0.086
FBG level (mg/dl)	0.04	0.02	0.024	-0.56	0.19	0.006	0.56	0.19	0.006	-0.02	0.01	0.008

CE coefficient, SE standard error

## Discussion

It is apparent from the present results that rise in blood glucose level, within physiological range, is associated augmented parasympathetic CAM, as indicated by higher values of LnHF and HF Norm. This finding is further supported by simultaneous lower readings of LF Norm and LnLF/HF, which point to sympathetic withdrawal at the upper limits of normal glycemic control. Previous researches exploring the effects of blood glucose concentration on HRV were mostly conducted among diabetic patients [13, 15, 22, 23] or other non-physiological conditions [24, 25], which explain why the present findings are probably the first to be reported. In contrast to our results, previous studies repeatedly demonstrated depressed HRV secondary to sympathetic predominance is cases with hyperglycemia [13, 15, 22, 23].

A previous study investigated glycemic threshold for impaired autonomic control gave conclusions in favor of the present findings, though it was based on different methodological technique [26]. Based on the results of baroreflex sensitivity, the study concluded that depressed autonomic control associated with high normal levels of glucose are due to aging, obesity and elevated blood pressure but never glucose concentration [26]. In another study, power spectral analysis of HRV during oral glucose tolerance test (OGTT) was investigated in 12 and 15 pregnant women with and without gestational diabetes mellitus (GDM) respectively [24]. Acute rise of blood glucose levels during the OGTT significantly activate sympathetic and depress parasympathetic discharge; however, the autonomic alterations were less marked in women with GDM compared with the controls. The same findings were reproduced when the effect of OGTT on sympathovagal balance was assessed in 30 healthy centenarians and 25 aged subjects [25].

A retrospective cohort investigating youth with insulin dependent diabetes mellitus showed that for every 1% increase in hemoglobin A1C over 6 years, there was a 5% reduction in the SD of the normal RR interval [22]. In addition, there was progressive decrease in HF Norm and increase in LF Norm independent of demographic, anthropometric, and cardiovascular risk factors [23]. In cases of hyperglycemia, glucose level can directly or indirectly, by inducing insulin release, influence cardiac autonomic modulations [13, 26]. The results of Bellavere et al showed attenuated HF bands and higher LF/HF ratio following insulin infusion in euglycaemic clamp model, suggesting negative feedback of insulin on parasympathetic discharge to the heart [27]. This implication was confirmed by Borne et al, who showed significant accentuation in muscle sympathetic nerve activities occurring simultaneously with euglycaemic hyperinsulinemic glucose clamp [15]. Similar results were obtained when cardiac autonomic modulations were evaluated in 18 diabetic patients and 8 age-matched healthy controls during hyperinsulinemic-hypoglycemic clamp [16]. Hypoglycemia was associated with higher MHR, but lower HF band, in the diabetic patients as well as the controls. According to another report, vagal withdrawal and augmented sympathetic activities are common during periods of hypoglycemia regardless of insulin level [17]. It is apparent from the above-mentioned reports that pattern of change in CAM during hypo- and hyperglycemia is comparable, being towards sympathetic predominance in the two extremes of glucose levels. According to the present results, sympathetic modulation, as indicated by LnLF/HF, was maximum in the lower FBG quintile and decreased gradually thereafter. The results also demonstrated gradual decrease in LnHF and HF Norm concurrently with the increase in LF Norm and LnLF/HF in the upper two FBG quintiles; however, Post Hoc analysis failed to document statistical significant for this rise. Accordingly, the enhanced sympathetic discharge associated with hypoglycemia seems to extend to the lower range of normal FBG, but decrease gradually and replaced by enhanced parasympathetic modulations at higher levels of normal FBG. Further researches are needed to set out the glycemic threshold beyond which further increase in glucose level readjusts sympathovagal balance towards sympathetic predominance again.

Limitations of this study include lack of insulin measurements among the young adults we studied. Combined evaluation of fasting insulin and glucose concentrations is likely to offer better background on insulin resistance among the studied subjects [28], and will clarify whether insulin or glucose level has more impact on cardiac autonomic modulations [15, 27]. Moreover, extending the FBG levels we studied to the diabetic range could have offer better chance for detection of the glycemic threshold beyond which higher glucose level is associated with sympathetic predominance. Larger sample size and adjustment for potential confounding factors like dietary habits and family history of heart problems should also be considered in future studies.

## Conclusion

The present study is the first to demonstrate that rise of blood glucose concentration, within physiological range, is associated with higher parasympathetic, but lower sympathetic CAM. Combining results of previous reports and the present, the enhanced sympathetic discharge associated with hypoglycemia seems to extend to the lower range of normal FBG, but decrease gradually and replaced by enhanced parasympathetic modulations at higher normal level of FBG. Further researches are needed to set out the glycemic threshold beyond which further increase in glucose level readjusts sympathovagal balance towards sympathetic predominance again.

## Supporting Information

S1 Raw data (DOCX)
