# Supplementary material for: Effect of Fasting Blood Glucose Level on Heart Rate Variability of Healthy Young Adults
Source: PLoS One. 2016 Jul 21;11(7):e0159820. doi: 10.1371/journal.pone.0159820 (PMC4956167; doi:10.1371/journal.pone.0159820)
Supplement: S1 Raw data — (DOCX) [file pone.0159820.s001.docx]

Raw Data

| Number | Gender | Age | Weight | Height | BMI | SBP | DBP | MABP | FBS | Quintiles | LnTP | LnVLF | LnLF | LnHF | LFNorm | HFNorm | LnLFHFR |
| --- | --- | --- | --- | --- | --- | --- | --- | --- | --- | --- | --- | --- | --- | --- | --- | --- | --- |
| 1 | Male | 21 | 56 | 177 | 17.9 | 100 | 60 | 73.3 | 60 | 1 | 7.14 | 5.84 | 6.32 | 5.90 | 60.10 | 39.90 | 0.41 |
| 2 | Female | 27 | 65.9 | 162 | 25.3 | 130 | 60 | 83.3 | 60 | 1 | 5.50 | 4.89 | 4.17 | 3.85 | 58.00 | 42.00 | 0.34 |
| 3 | Male | 30 | 71.5 | 173 | 23.9 | 130 | 90 | 103 | 60 | 1 | 4.10 | 3.50 | 2.97 | 2.01 | 72.10 | 27.90 | 0.96 |
| 4 | Male | 29 | 79.2 | 165 | 29.3 | 130 | 80 | 96.7 | 64 | 1 | 5.40 | 4.74 | 4.37 | 3.31 | 74.30 | 25.70 | 1.06 |
| 5 | Female | 28 | 92.7 | 163 | 35.1 | 122 | 82 | 95.3 | 67 | 1 | 6.89 | 6.19 | 5.92 | 4.84 | 74.60 | 25.40 | 1.06 |
| 6 | Female | 33 | 71.5 | 156 | 29.6 | 120 | 80 | 93.3 | 67 | 1 | 6.54 | 5.50 | 5.68 | 5.04 | 65.60 | 34.40 | 0.64 |
| 7 | Male | 25 | 41.5 | 161 | 16 | 110 | 70 | 83.3 | 67 | 1 | 5.16 | 4.60 | 4.09 | 2.71 | 79.80 | 20.20 | 1.36 |
| 8 | Female | 22 | 36.8 | 158 | 14.8 | 130 | 70 | 90 | 67 | 1 | 6.05 | 4.28 | 5.00 | 5.33 | 41.80 | 58.20 | -0.36 |
| 9 | Female | 29 | 67 | 161 | 26 | 120 | 80 | 93.3 | 67 | 1 | 5.71 | 5.42 | 4.06 | 2.92 | 75.60 | 24.40 | 1.13 |
| 10 | Male | 23 | 47.5 | 166 | 17.3 | 100 | 60 | 73.3 | 73 | 2 | 7.97 | 5.45 | 7.44 | 6.87 | 63.90 | 36.10 | 0.59 |
| 11 | Male | 29 | 73.8 | 168 | 26.1 | 124 | 70 | 88 | 73 | 2 | 5.16 | 3.98 | 4.05 | 4.14 | 47.80 | 52.20 | -0.11 |
| 12 | Female | 21 | 63.7 | 159 | 25.4 | 135 | 75 | 95 | 73 | 2 | 5.53 | 4.59 | 4.83 | 3.35 | 81.40 | 18.60 | 1.48 |
| 13 | Female | 21 | 73.4 | 161 | 28.5 | 105 | 70 | 81.7 | 73 | 2 | 8.19 | 6.55 | 6.29 | 7.77 | 18.60 | 81.60 | -1.61 |
| 14 | Female | 33 | 62.7 | 165 | 23.2 | 100 | 70 | 80 | 80 | 2 | 6.71 | 6.45 | 4.93 | 3.86 | 74.40 | 25.60 | 1.06 |
| 15 | Female | 25 | 42.3 | 166 | 15.4 | 110 | 75 | 86.7 | 85 | 2 | 8.25 | 6.28 | 7.02 | 7.69 | 33.90 | 66.10 | -0.69 |
| 16 | Male | 21 | 61.1 | 175 | 20 | 120 | 70 | 86.7 | 89 | 2 | 9.51 | 9.17 | 7.41 | 7.74 | 41.80 | 58.20 | -0.36 |
| 17 | Female | 20 | 63 | 167 | 22.7 | 120 | 80 | 93.3 | 89 | 2 | 7.79 | 5.94 | 6.58 | 7.17 | 35.60 | 64.40 | -0.51 |
| 18 | Female | 21 | 68 | 166 | 24.7 | 140 | 90 | 107 | 89 | 2 | 6.04 | 5.05 | 5.32 | 4.09 | 77.50 | 22.50 | 1.22 |
| 19 | Male | 21 | 53 | 168 | 18.9 | 110 | 70 | 83.3 | 93 | 3 | 6.58 | 5.87 | 5.48 | 4.83 | 65.60 | 34.40 | 0.64 |
| 20 | Female | 30 | 109 | 161 | 41.9 | 120 | 80 | 93.3 | 93 | 3 | 4.72 | 4.41 | 2.63 | 2.81 | 45.60 | 54.40 | -0.22 |
| 21 | Male | 28 | 74.8 | 168 | 26.5 | 120 | 70 | 86.7 | 95 | 3 | 6.58 | 6.23 | 4.92 | 4.30 | 64.90 | 35.10 | 0.64 |
| 22 | Male | 27 | 68.9 | 171 | 23.6 | 110 | 70 | 83.3 | 95 | 3 | 8.73 | 7.52 | 7.60 | 7.76 | 45.90 | 54.10 | -0.22 |
| 23 | Male | 22 | 83.5 | 184 | 24.7 | 120 | 80 | 93.3 | 95 | 3 | 6.64 | 4.67 | 5.20 | 6.16 | 27.70 | 72.30 | -0.92 |
| 24 | Male | 28 | 77.7 | 170 | 26.9 | 120 | 75 | 90 | 95 | 3 | 9.16 | 7.02 | 7.98 | 8.61 | 34.70 | 65.30 | -0.69 |
| 25 | Male | 21 | 89.1 | 177 | 28.4 | 130 | 90 | 103 | 98 | 4 | 5.11 | 3.54 | 4.04 | 4.32 | 43.00 | 57.00 | -0.22 |
| 26 | Female | 20 | 73 | 164 | 27.1 | 100 | 60 | 73.3 | 98 | 4 | 6.07 | 4.85 | 5.19 | 4.83 | 59.00 | 41.00 | 0.34 |
| 27 | Male | 35 | 58 | 174 | 19.1 | 110 | 70 | 83.3 | 98 | 4 | 8.80 | 6.42 | 7.77 | 8.21 | 39.20 | 60.80 | -0.51 |
| 28 | Female | 25 | 62.5 | 155 | 26.2 | 110 | 65 | 80 | 100 | 5 | 5.99 | 4.18 | 3.95 | 5.64 | 15.50 | 84.50 | -1.61 |
| 29 | Male | 27 | 73.1 | 168 | 25.9 | 110 | 75 | 86.7 | 100 | 5 | 6.71 | 5.57 | 5.87 | 5.29 | 64.10 | 35.90 | 0.59 |
| 30 | Male | 23 | 54.3 | 181 | 16.7 | 170 | 70 | 103 | 100 | 5 | 7.24 | 6.05 | 6.51 | 5.70 | 69.20 | 30.80 | 0.79 |
| 31 | Male | 23 | 71 | 175 | 23.3 | 130 | 80 | 96.7 | 100 | 5 | 8.89 | 7.65 | 7.72 | 7.98 | 43.40 | 56.60 | -0.22 |
| 32 | Male | 21 | 64 | 180 | 19.9 | 110 | 70 | 83.3 | 100 | 5 | 6.81 | 6.01 | 5.60 | 5.41 | 54.70 | 45.30 | 0.18 |
| 33 | Male | 20 | 60 | 176 | 19.5 | 110 | 70 | 83.3 | 100 | 5 | 8.33 | 7.01 | 7.01 | 7.57 | 36.40 | 63.60 | -0.51 |
| 34 | Male | 28 | 62.3 | 170 | 21.7 | 110 | 70 | 83.3 | 100 | 5 | 6.67 | 6.29 | 5.03 | 4.52 | 62.30 | 37.70 | 0.53 |
| 35 | Male | 24 | 108 | 177 | 34.3 | 160 | 90 | 113 | 100 | 5 | 6.06 | 5.49 | 4.98 | 3.66 | 79.00 | 21.00 | 1.34 |
| 36 | Male | 24 | 85 | 172 | 28.9 | 110 | 90 | 96.7 | 100 | 5 | 7.28 | 5.54 | 6.61 | 6.12 | 62.00 | 38.00 | 0.47 |
| 37 | Female | 30 | 61.4 | 160 | 23.9 | 120 | 80 | 93.3 | 100 | 5 | 6.54 | 5.34 | 5.04 | 5.79 | 32.00 | 68.00 | -0.69 |
| 38 | Female | 27 | 55.2 | 159 | 21.8 | 130 | 80 | 96.7 | 100 | 5 | 6.97 | 5.23 | 6.08 | 6.09 | 49.60 | 50.40 | 0.00 |
| 39 | Female | 37 | 88 | 168 | 31.2 | 130 | 90 | 103 | 100 | 5 | 6.54 | 5.25 | 5.45 | 5.60 | 46.20 | 53.80 | -0.11 |
| 40 | Female | 38 | 57.3 | 160 | 22.3 | 120 | 90 | 100 | 100 | 5 | 4.86 | 3.49 | 3.43 | 4.19 | 32.00 | 68.00 | -0.69 |
| 41 | Female | 20 | 47 | 158 | 18.8 | 120 | 80 | 93.3 | 100 | 5 | 4.91 | 3.15 | 3.91 | 4.15 | 44.00 | 56.00 | -0.22 |
| 42 | Male | 28 | 89.2 | 174 | 29.4 | 110 | 80 | 90 | 100 | 5 | 6.48 | 6.01 | 5.24 | 3.98 | 78.10 | 21.90 | 1.28 |

BMI = body mass index; SBP = Systolic blood pressure; DBP = diastolic blood pressure; MABP = mean arterial blood pressure; FBS fasting blood glucose, Ln = natural logarithm; TP = total power; VLF = very low frequency; LF = low frequency; HF = high frequency; LF/HF = low frequency/ high frequency ratio; LF Norm = normalized low frequency; HF Norm = normalized high frequency.
